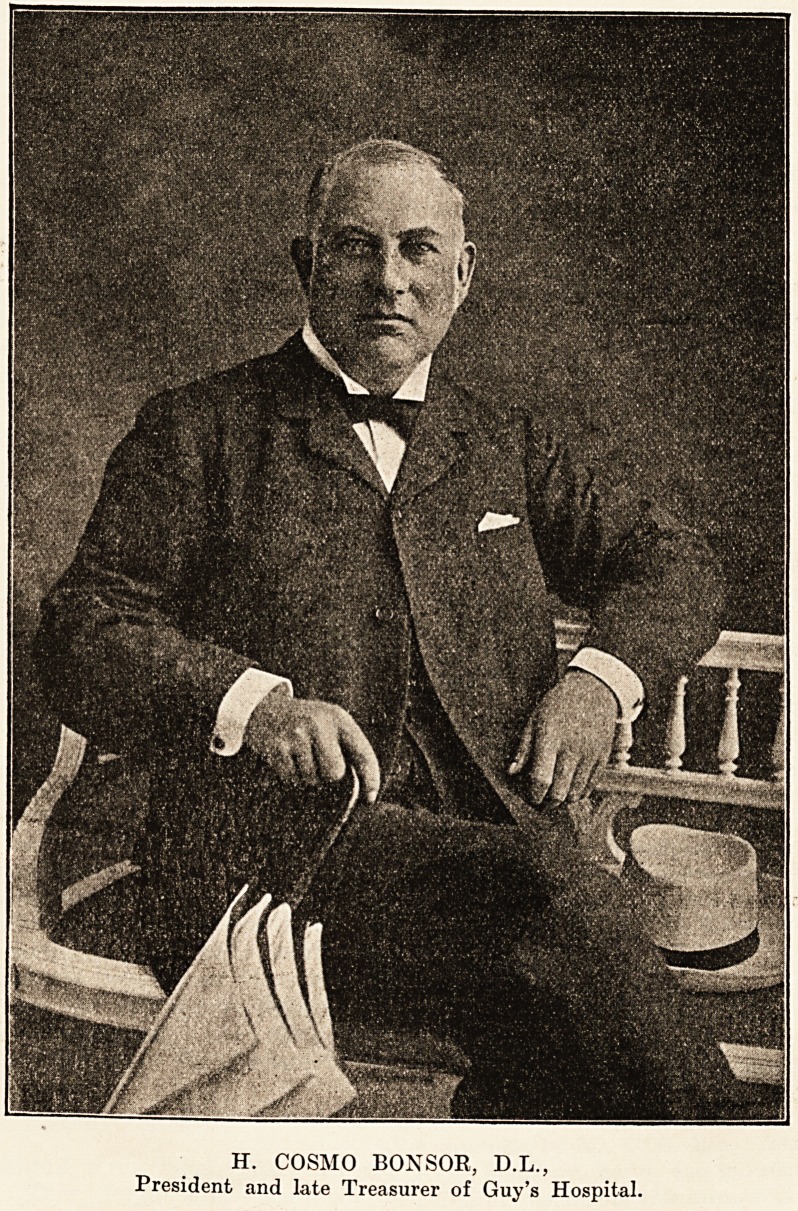# Eminent Chairman Series

**Published:** 1910-12-10

**Authors:** 


### SPECIAL INSTITUTIONAL ARTICLES.

### EMINENT CHAIRMAN SERIES.

### III.—MR. COSMO BONSOR, D.L., PRESIDENT AND LATE TREASURER OF GUY'S HOSPITAL, LONDON.

Mr. Henry Cosmo Bonsor, D.L., who is Chairman of the South Eastern Railway and a Director of the Bank of England, is one of the best known and most widely respected and liked men of business in the City of London. He was elected a Governor of Guy's Hospital in 1882, and appointed Treasurer in 1896 in circumstances which are remarkable in the history of voluntary hospitals. Since Thomas Guy founded the hospital it has been served by twelve treasurers, and of these the portraits of two only, Mr. Harrison and Mr. Thomas Turner, keep company in the court-room of the hospital with that of the founder. Harrison and Turner were what are known at Guy's Hospital as great treasurers. They maintained a high standard of administration in time of prosperity, a rare virtue in the history of all endowed institutions, and their names are, and always will be, remembered with gratitude by everyone interested in Guy's. The Governors of Guy's Hospital last year spontaneously expressed the unanimous feeling that Mr. Bonsor should allow his portrait to be painted and fixed in a panel in the court-room of the hospital. Subsequently, on the death of King Edward, the office of President of Guy's Hospital became vacant, and Mr. Cosmo Bonsor was justly honoured by being elected to fill this great post.

H. COSMO BONSOR, D.L.,
President and late Treasurer of Guy's Hospital.

The year 1896 must ever stand out as momentous in the history of Guy's. At that time the hospital was languishing for want of funds, which resulted in a hundred and fifty of its beds being closed to the sick poor, but to make matters worse, as the late Mr. W. E. Gladstone stated in a speech he made in support of the hospital, "its revenue, that was over £40,000, had sunk to £20,000 per annum." The trouble was to find some means of overcoming this financial collapse, due to causes over which the Governors could exercise no control. The late King Edward VII., then Prince of Wales, was invited to preside at a public dinner with the object of bringing all the facts to the notice of the public. The Prince of Wales took a great deal of personal trouble, went thoroughly into the position of the hospital, and decided that before he could consent to preside at such a dinner he must have guarantees that certain necessary changes would be made, that a carefully thought out and organised scheme for raising the necessary money should be created, and that the right man should be found to take up the duties of treasurer with zeal and energy. Everybody connected with Guy's Hospital showed the warmest appreciation of the genuine interest thus displayed by the Prince of Wales; all personal feelings were sunk and difficulties at once removed, and everyone, including the honorary medical staff, from the then president, the late Lord Aldenham, and the then treasurer, Mr. E. H. Lushington, set to work with a will to make this dinner memorable in English history.

The story of that dinner has been told in these columns already, and everyone interested can find the details on referring to The Hospital for June 20, 1896, pages 196 *et seq.* After much anxious consideration the Governors determined to invite Mr. Cosmo Bonsor to become treasurer of the hospital, and he at once set to work practically to rebuild and to re-endow Guy's Hospital to the extent of the loss sustained from the reduction of the rent-roll from the estates. The festival dinner, at which the Prince of Wales presided, at the Imperial Institute, produced the largest sum ever raised for a hospital on such an occasion: £172,000, in round figures, was received and promised, including an additional revenue in annual subscriptions of £3,300 a year. This was a substantial send-off, and by steady, persistent, quiet work Mr. Cosmo Bonsor obtained from voluntary sources during the fifteen years of his treasurership, *i.e.* from 1896 to 1910, a re-endowment fund in invested securities to the amount of £310,000, and an increase in the average income of the hospital from all sources, including donations, subscriptions, legacies, invested property (estates), dividends and investments, and miscellaneous receipts of £52,700 per annum. This large addition to revenue represented an actual increase in the average income of the hospital from all sources equal, in round figures, to 120 per cent., compared with that for the ten years prior to Mr. Bonsor's treasurership—namely, from April 1886 to April 1896. This record of successful hospital finance is as unique as it is remarkable.

### A New Hospital.

Mr. Cosmo Bonsor's treasurership resulted in the production of what practically amounts to a new hospital, so far as the buildings, equipment, and organisation of its many departments are concerned. The first practical result of his work was the reopening and maintenance of the hundred and fifty beds previously closed to the sick poor for lack of funds. He then took in hand the completion, renovation, and readaptation of all the wards and the various departments of the hospital, and brought each and all of them up to a high state of efficiency. The usefulness of the buildings devoted to the reception of patients was made complete by the erection of new open-air balconies and sanitary blocks to all wards. Four new operation theatres were provided in the surgical buildings, and three new operation theatres in the medical section of the hospital. A new isolation block was erected; a new and complete laundry, capable of dealing with the whole of the washing of the institution, was provided; a central station for the supply of light, water, heat, and power throughout the entire hospital buildings was established; and the Henriette Raphael Nurses' Home, one of the most beautiful residences for nurses in the country, which contains a large swimming-bath, was erected and opened. Guy's Hospital nurses have been fortunate to be privileged to work under such a treasurer. He has ever been anxious to promote everything which could minister to their comfort and advantage. Several prizes and medals have been founded for nurses, including the Cazenove Prize, the Butterworth Medal, the Keogh Prize, and the Raphael Massage Prize, and the Raphael Nurses' Fund. Those members of the staff who have preferred to devote themselves to private nursing and have joined Guy's Hospital Trained Nurses' Institution, are amongst the most fortunate members of their profession. The whole of the net earnings of the Nurses' Institution (with the exception of a small sum yearly reserved for emergencies) is devoted to the benefit of the nursing staff. Every nurse joins the National Pension Fund, and the Institution contributes liberally every year to this fund in co-operation with the nurse to enable her to obtain a pension at the age of fifty years. At the end of each year the treasurer of Guy's Hospital takes such a sum as he deems desirable from the profits of the Institution and applies it upon an equitable sliding-scale as the bonus fund, and all nurses in their fifth year of service receive proportionate benefit. Finally, a new out-patient department, well planned and efficiently and thoroughly equipped, was built. It is estimated that the carrying out of all these important works has entailed an expenditure of quite £400,000, a sum which would have been greatly increased had it not been for the strict economy enforced under a model system of control, which was fully explained on pages 189 *et seq* of The Hospital for November 16, 1907.

### New Departments of Medical Work.

Mr. Cosmo Bonsor has proved himself to be one of the most enterprising, far-seeing, and up-to-date hospital treasurers. Not only did he set himself to put the finances into a thoroughly satisfactory condition, to reconstruct and practically rebuild the whole institution, but he made it his business to co-operate heartily with the honorary medical staff, and so to secure that necessary new departments of medical work should be introduced or extended, so that Guy's Hospital might be kept thoroughly abreast of every modern and scientific development. These new departments of medical work include the following: The actino-therapeutic department, the bacteriological department, the genito-urinary department, the massage department, the nervous diseases department, the orthopædic department, the photographic department, the skiagraphic department, and a department for diseases of the throat.

### The Medical School.

Great developments have taken place in the medical school. Two large and commodious lecture-theatres, a beautifully appointed medical library, and many new class-rooms have been fitted up with money specially subscribed for the purpose. Several additional scholarships and prizes have been founded for students—namely, the Gordon Lectureship, £250; the Arthur Durham Travelling Studentship, about £100; the Charles Oldham Prize of £30; the Newland-Pedley Prize, gold medal; the Greville Research Studentship, £200; and the Douglas Research Studentship, £300.

### An Appreciation.

A gentleman who has been intimately associated with Mr. Cosmo Bonsor during the whole of his treasurership of Guy's Hospital writes:—

"Guy's Hospital has had great treasurers in the past, who maintained a high standard of administration in times of prosperity—blessed be their names; but one's heart goes out to the man who, in the day of great adversity, unselfishly devoted to the cause of the hospital his means, time, and capacity, his great influence, his wise powers of control and administration, and last, but not least, his cheery strength and kindliness." Such a man is Mr. Cosmo Bonsor, as they have found him at Guy's Hospital. "There is no doubt about the genuine esteem and regard in which Mr. Bonsor is held by all who have known him at Guy's, and throughout his treasurership his spirit has been a force stimulating his co-workers of every degree to join in one splendid and successful effort to restore the fortunes of the hospital. Besides being a generous donor to its funds Mr. Bonsor was always foremost in the support of every good subsidiary work, whether initiated by the Governors, medical staff, students, or the nurses, and such activities were numerous. Mr. Bonsor's devotion to every interest has welded together all the departments of the hospital, including the medical and dental schools, and he has left to the care of his successor a vast institution in which all sections of men and things are working in one harmonious whole."

This record of one great hospital treasurer's continuous voluntary labours for fifteen consecutive years must win the admiration and should stimulate the emulation of voluntary hospital workers all the world over.

## Figures and Tables

**Figure f1:**